# Impact of 3D Virtual Modeling on Perioperative Outcomes in Robot-Assisted Partial Nephrectomy

**DOI:** 10.3390/diagnostics16071082

**Published:** 2026-04-03

**Authors:** Francesco Passaro, Achille Aveta, Gianluca Spena, Antonio Tufano, Savio Domenico Pandolfo, Giovanni Grimaldi, Dario Franzese, Luigi Castaldo, Giuseppe Quarto, Eleonora Monteleone, Laura Brunella Alfè, Giovanna Canfora, Sonia Desicato, Antonio Scarpato, Raffaele Muscariello, Alessandro Izzo, Roberto Contieri, Sisto Perdonà

**Affiliations:** 1 Department of Urology, National Cancer Institute IRCCS G. Pascale Foundation, 80131 Naples, Italy; francescopassaro1996@gmail.com (F.P.); spena.dr@gmail.com (G.S.); g.grimaldi@istitutotumori.na.it (G.G.); dario.franzese@istitutotumori.na.it (D.F.); l.castaldo@istitutotumori.na.it (L.C.); g.quarto@istitutotumori.na.it (G.Q.); eleonora.monteleone@istitutotumori.na.it (E.M.); laurabrunella.alfe@istitutotumori.na.it (L.B.A.); s.desicato@istitutotumori.na.it (S.D.); r.muscariello@istitutotumori.na.it (R.M.); dott.alessandro.izzo@gmail.com (A.I.); contieri.ro@gmail.com (R.C.); s.perdona@istitutotumori.na.it (S.P.); 2 Department of Urology, San Carlo di Nancy Hospital, 00165 Rome, Italy; antonio.tufano91@gmail.com; 3 Department of Life, Health and Environmental Sciences, University of L’Aquila, 67100 L’Aquila, Italy; pandolfosavio@gmail.com; 4 Department of Neurosciences, Science of Reproduction and Odontostomatology, University of Naples Federico II, 80131 Naples, Italy; antonioscarpato1992@gmail.com; 5 Division of Anesthesia and Pain Medicine, Istituto Nazionale Tumori IRCCS Fondazione Pascale, 80131 Naples, Italy; giovanna.canfora@istitutotumori.na.it

**Keywords:** robot-assisted partial nephrectomy, 3D virtual modeling, surgical navigation, renal cell carcinoma

## Abstract

**Background/Objectives:** Robot-assisted partial nephrectomy (RAPN) remains a technically demanding procedure, associated with a non-negligible risk of perioperative complications. This study aimed to assess the impact of preoperative planning and intraoperative navigation using patient-specific three-dimensional (3D) virtual model reconstructions on perioperative outcomes of RAPN. **Methods:** We analyzed 307 patients who underwent RAPN for renal tumors at a tertiary center between 2021 and 2024. Starting in 2023, 3D modeling (Medics3D) was integrated for selected cases (*n* = 69) and compared to a 2D-imaging control group (*n* = 238). The primary outcome was trifecta achievement, defined as the simultaneous presence of negative surgical margins, ≥90% preservation of preoperative eGFR at discharge, and absence of perioperative complications. Clamping strategies were categorized as on-clamp, selective/super-selective, or off-clamp. Mann–Whitney and Chi-squared tests compared the groups; multivariable logistic regression identified independent predictors of trifecta achievement. **Results:** Baseline characteristics were balanced between the 3D and control groups: median age (62 vs. 61 years, *p* = 0.5), BMI (28 vs. 26, *p* = 0.3), and eGFR (85 vs. 86 mL/min/1.73 m^2^, *p* = 0.5). Median tumor size was 4.2 vs. 4.0 cm (*p* = 0.4), and RENAL complexity was comparable (*p* = 0.12). Selective or super-selective clamping was significantly more frequent in the 3D group (32% vs. 15%; *p* < 0.01). While WIT (17.5 vs. 18.5 min, *p* = 0.09) and complication rates (26% vs. 29%, *p* = 0.7) were similar, the 3D group showed a significantly lower rate of positive surgical margins (5% vs. 15%; *p* = 0.030). Trifecta achievement was significantly higher in the 3D group (51% vs. 32%; *p* = 0.004). On multivariable analysis, 3D modeling remained an independent predictor of trifecta achievement (OR 2.1, 95% CI 1.17–3.70; *p* = 0.013). **Conclusions:** The use of patient-specific 3D kidney reconstructions was associated with improved perioperative outcomes in patients undergoing RAPN. These findings support the integration of 3D modeling into routine surgical workflows to enhance operative precision and optimize patient outcomes.

## 1. Introduction

Robot-assisted partial nephrectomy (RAPN) has progressively become the preferred surgical approach for the management of clinical T1 renal masses, largely due to its minimally invasive nature and its ability to combine oncological efficacy with optimal renal function preservation [1,2,3,4]. Compared with open and conventional laparoscopic techniques, RAPN offers enhanced dexterity, tremor filtration, and superior visualization, allowing surgeons to perform precise tumor excision while minimizing ischemic injury to the remaining parenchyma [5,6]. These advantages have contributed to its widespread adoption in high-volume centers and its endorsement by contemporary international guidelines [7,8].

Despite these technical improvements, RAPN remains a complex and highly operator-dependent procedure. Surgical difficulty is influenced by tumor size, depth, location, and proximity to critical anatomical structures such as segmental arteries and the collecting system [9]. Achieving an optimal balance between complete oncological resection and maximal functional preservation requires meticulous planning and accurate intraoperative anatomical orientation [6,10,11].

Preoperative imaging plays a pivotal role in surgical planning for RAPN. Conventional imaging modalities, including contrast-enhanced computed tomography (CT) and magnetic resonance imaging (MRI), provide detailed anatomical information; however, their two-dimensional representation inherently limits the surgeon’s ability to fully appreciate the complex three-dimensional relationships within the kidney. Translating axial, coronal, and sagittal images into a comprehensive mental 3D map remains cognitively demanding and subject to interobserver variability, particularly in anatomically complex tumors [12].

**Figure 1:**
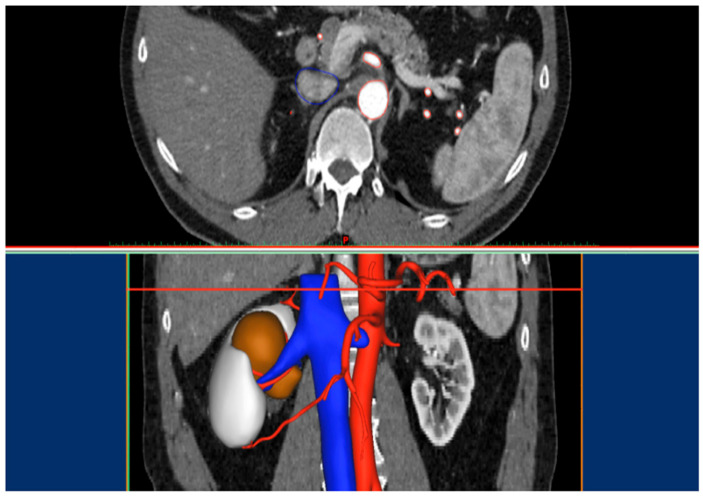
CT-based vascular segmentation (**upper section**) and subsequent three-dimensional volumetric reconstruction (**lower section**).

In this context, patient-specific three-dimensional (3D) virtual reconstructions have emerged as a promising adjunct to standard imaging [13,14]. By converting conventional radiological datasets into individualized volumetric models, 3D reconstructions provide an intuitive visualization of renal anatomy, including the tumor, arterial and venous branches, and the collecting system (Figure 1). This enhanced spatial understanding may facilitate surgical decision-making, enabling more accurate identification of tumor-feeding vessels, improved planning of resection planes, and refinement of clamping strategies tailored to the specific anatomy of each patient [14,15].

Beyond preoperative planning, 3D models can also be integrated into intraoperative navigation systems, allowing real-time reference during tumor excision and reconstruction. This integration has the potential to reduce uncertainty during critical operative steps and to improve consistency in surgical performance [11,15]. Recent technological advancements have further expanded the capabilities of 3D modeling. For example, anatomical digital twins incorporating texture simulation and machine-learning–based algorithms have demonstrated superiority over conventional 3D reconstructions in identifying vascular structures and tumor boundaries, thereby enhancing anatomical comprehension [16].

Additionally, meta-analyses comparing 3D-assisted and conventional minimally invasive partial nephrectomy have reported significant reductions in transfusion rates and collecting system openings, suggesting a tangible impact on perioperative morbidity [14]. Nevertheless, the existing literature remains heterogeneous. While some studies have reported improvements in perioperative outcomes and surgical precision, others have failed to demonstrate significant benefits in terms of positive surgical margins or postoperative renal function preservation, highlighting the influence of study design, patient selection, tumor complexity, and degree of workflow integration.

Emerging 3D platforms incorporating vascular perfusion and ischemia-mapping algorithms have shown promising results in guiding super-selective clamping strategies with the aim of maximizing nephron preservation; however, robust clinical validation remains limited [17]. Moreover, the role of 3D technologies is expanding beyond clinical application into surgical education and training, where virtual and physical models have been shown to enhance anatomical understanding and procedural planning, particularly for complex renal masses [18].

Given this evolving landscape, further high-quality clinical studies are required to delineate the true impact of 3D-assisted planning and navigation on perioperative outcomes in RAPN. In particular, composite outcome measures such as trifecta achievement, which integrates oncological safety, functional preservation, and perioperative morbidity, may provide a more comprehensive assessment of surgical quality. The present study aims to evaluate the effect of patient-specific 3D virtual reconstructions on perioperative outcomes of RAPN within a large single-center cohort, reflecting the real-world integration of this technology into routine clinical practice.

## 2. Materials and Methods

### 2.1. Study Design

We retrospectively analyzed data from 307 consecutive patients who underwent RAPN for renal tumors at a tertiary referral center in Italy between January 2021 and December 2024. The study population included patients treated in a real-world clinical setting, reflecting routine surgical practice at a high-volume robotic center. This retrospective observational study was conducted using fully anonymized data collected for clinical and quality-assurance purposes. According to institutional policies and national regulations, formal ethical committee approval was not required, as all patients included were already enrolled in the ONCOCAMP research protocol.

Beginning in January 2023, patient-specific 3D virtual reconstructions were progressively introduced into the institutional surgical workflow for selected patients undergoing RAPN. We included all adult patients (>18 years) with cT1-T2 renal masses who underwent RAPN. Exclusion criteria were: multiple ipsilateral tumors, solitary kidney, or poor-quality preoperative imaging that precluded 3D reconstruction. The decision to use 3D modeling was based on availability of the technology and surgeon preference, particularly in cases perceived as anatomically complex. Patients who underwent preoperative planning and intraoperative navigation with 3D virtual models developed using Medics3D software (Medics S.r.l., Turin, Italy) constituted the intervention group (3D group). Patients treated during the same study period using conventional two-dimensional imaging modalities alone formed the control group.

The selection of patients for 3D-assisted planning was primarily based on the availability of the technology and surgeon preference, with priority given to anatomically complex cases or those involving hilar or endophytic tumors. Not all eligible patients could undergo 3D modeling because of logistical factors such as scheduling constraints or temporary unavailability of the segmentation team. Although this could theoretically introduce selection or temporal bias, the balance of baseline variables and the inclusion of the RENAL complexity score in multivariable analyses were intended to mitigate these effects. Because the 3D modeling program was implemented in the later phase of the study period, all surgeons performing RAPN had already completed their institutional learning curve and were operating under stable perioperative protocols, minimizing variability due to evolving experience or workflow changes.

All surgical procedures were performed by experienced robotic surgeons with extensive expertise in RAPN (more than 5 years of robotic practice and over 500 robotic-assisted partial nephrectomies performed before 2021), minimizing variability related to the learning curve. No experimental interventions were applied, and all patients received standard-of-care surgical treatment according to institutional protocols.

The primary outcome was trifecta achievement, defined as the simultaneous presence of negative surgical margins, preservation of at least 90% of the preoperative estimated glomerular filtration rate (eGFR) at hospital discharge, and the absence of perioperative complications. Secondary outcomes included operative time, warm ischemia time (WIT), estimated blood loss (EBL), major complications, and length of hospital stay (LOS).

All patients underwent RAPN using a standardized transperitoneal approach and the Da Vinci X robotic system. Patient positioning, port placement, and docking were performed according to institutional protocols and surgeon preference, ensuring consistency across cases. Tumor excision and renal reconstruction were carried out to optimize precision.

**Figure 2:**
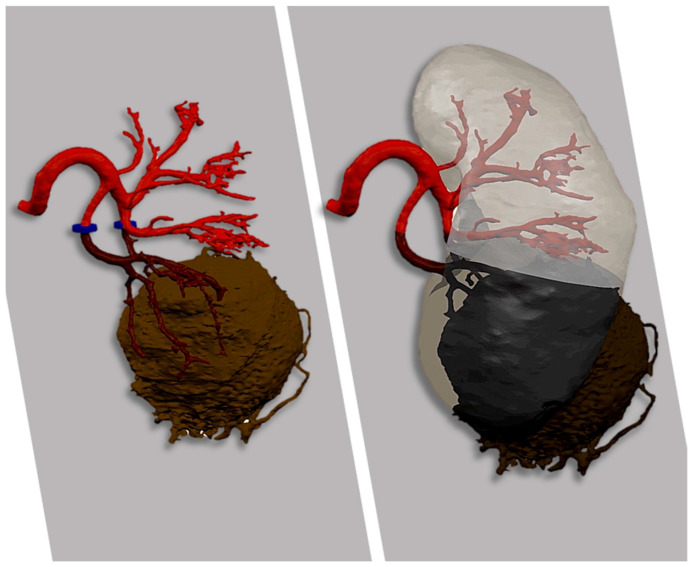
Selective renal artery clamping (**left**) and the resulting renal ischemia induced by vascular clamping (**right**).

Renal artery clamping was applied based on tumor characteristics and anticipated bleeding risk, explicitly distinguishing between: (1) on-clamp (main renal artery), (2) selective or super-selective clamping (segmental or subsegmental branches), and (3) off-clamp. When anatomically feasible, selective strategies were favored to minimize global renal ischemia and maximize functional preservation (Figure 2). The decision regarding clamping was made at the surgeon’s discretion, supported by preoperative imaging and intraoperative assessment. All procedures were performed using the Da Vinci X robotic surgical system (Intuitive Surgical, Sunnyvale, CA, USA).

### 2.2. Surgical Approach

**Figure 3:**
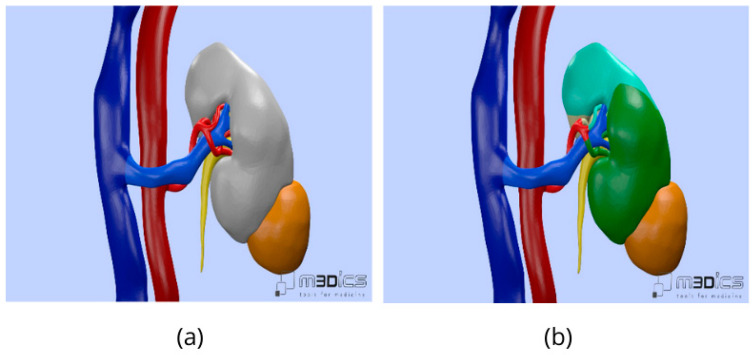
(**a**) Patient-specific three-dimensional reconstruction illustrating an exophytic lower-pole renal mass; (**b**) Segmental vascular mapping of the renal parenchyma displayed with a “rainbow” perfusion-based color model.

All patients underwent preoperative contrast-enhanced CT imaging as part of the standard diagnostic and staging workup. For patients in the 3D group, CT imaging datasets were processed using dedicated software to generate patient-specific virtual 3D kidney models. These models provided detailed visualization of renal parenchyma, tumor location and extent, arterial and venous anatomy, and the collecting system, allowing a comprehensive assessment of anatomical relationships (Figure 3).

### 2.3. Imaging and Tumor Complexity Assessment

Tumor complexity was evaluated using both the PADUA and RENAL nephrometry scoring systems. In the 3D group, nephrometry scores were assigned based on the virtual models and jointly reviewed by a trained urologist and a bioengineer to ensure accuracy and reproducibility. In the control group, nephrometry scores were calculated using conventional 2D imaging and independently assigned by two blinded urologists. Discrepancies were resolved by consensus.

### 2.4. Data Collection

Demographic, clinical, and perioperative data were collected from institutional electronic medical records. Baseline variables included age, body mass index (BMI), and preoperative estimated glomerular filtration rate (eGFR). Tumor-related variables included tumor size (maximum diameter in cm), pathological T-stage, and nephrometry scores (RENAL and PADUA).

We recorded the following perioperative and functional parameters to ensure consistency with clinical reporting: operative time (min), warm ischemia time (WIT, min), estimated blood loss (EBL, mL), and length of hospital stay (LOS, days). The clamping strategy was explicitly categorized as: (1) on-clamp (main renal artery), (2) selective or super-selective (segmental or subsegmental branches), and (3) off-clamp. Surgical safety was further assessed by the rate of conversion to radical nephrectomy and the occurrence of postoperative complications, which were graded according to the Clavien–Dindo classification. Renal function preservation was defined as retention of at least 90% of preoperative eGFR at the time of hospital discharge (Table 1).

**Table 1:** Baseline demographic, clinical, and tumor characteristics of the overall cohort and comparison between patients undergoing standard imaging versus 3D-assisted planning.

Characteristic	Overall (*N* = 307)	Control (*N* = 238)	3D Model (*N* = 69)	*p*-Value
Age, median (IQR)	61 (54–70)	61 (53–70)	62 (55–70)	0.5
BMI, median (IQR)	26 (24–30)	26 (24–30)	28 (24–31)	0.3
RENAL Complexity, *n* (%)				0.12
Low	223 (73%)	171 (71.85%)	52 (75.4%)	
Moderate	81 (26%)	66 (27.73%)	15 (21.7%)	
High	3 (1.0%)	1 (0.42%)	2 (2.9%)	
Pathological T Stage, *n* (%)				0.8
T1	204 (66.5%)	157 (66%)	47 (68.1%)	
T2	92 (30%)	73 (30.7%)	19 (27.5%)	
T3	9 (2.9%)	7 (2.9%)	2 (2.9%)	
T4	2 (0.6%)	1 (0.4%)	1 (1.5%)	
Preoperative eGFR, median (IQR)	85 (69–96)	86 (70–97)	85 (68–92)	0.5
Positive Surgical Margins (PSM), *n* (%) (*N* = 306)				0.03
Negative	268 (87%)	202 (85%)	66 (95%)	
Positive	39 (13%)	36 (15%)	3 (5%)	
eGFR Preservation ≥ 90%, *n* (%)				0.8
No	111 (36%)	87 (37%)	24 (35%)	
Yes	196 (64%)	151 (63%)	45 (65%)	
Postoperative Complications (All Clavien), *n* (%)				0.7
None	221 (72%)	170 (71%)	51 (74%)	
Present	86 (28%)	68 (29%)	18 (26%)	
Trifecta Achievement, *n* (%)				0.004
No	196 (64%)	162 (68%)	34 (49%)	
Yes	111 (36%)	76 (32%)	35 (51%)	
Tumor Size (cm), median (IQR)	4.1 (3.0–7.3)	4.0 (2.9–7.2)	4.2 (3.1–7.4)	0.4
Operative time (min), Median (IQR)	160 (140–185)	162 (142–188)	155 (138–180)	0.12
Warm ischemia time (min), Median (IQR)	18.2 (14–22)	18.5 (15–23)	17.5 (13–21)	0.09
Estimated blood loss (mL), Median (IQR)	145 (100–200)	150 (110–210)	130 (90–190)	0.2
Length of stay (days), Median (IQR)	3 (2–4)	3 (2–4)	3 (2–4)	0.7
Selective Clamping, *N* (%)	58 (19%)	36 (15%)	22 (32%)	<0.01

### 2.5. Statistical Analysis

Descriptive statistics were used to summarize patient characteristics and perioperative outcomes. Continuous variables were reported as medians with interquartile ranges (IQR) and compared between groups using the Mann–Whitney U test, given their non-normal distribution. Categorical variables were expressed as frequencies and percentages and compared using the Chi-squared test.

To identify independent predictors of trifecta achievement, univariate and multivariable logistic regression analyses were performed. Covariates included in the multivariable model (age, BMI, RENAL nephrometry score, and the use of 3D modeling) were selected a priori based on their clinical relevance and established significance in the existing literature. This approach ensured that the impact of 3D virtual reconstructions was appropriately adjusted for potential baseline confounders while preserving statistical power. Results were reported as odds ratios (ORs) with 95% confidence intervals (CIs).

Cases with incomplete data for individual outcomes (≤5%) were excluded from specific analyses but retained for the overall dataset. Model calibration and discrimination were assessed using Hosmer–Lemeshow and pseudo-R^2^ statistics. Although propensity score–based matching was not applied, the two groups were well-balanced with respect to demographic and clinical parameters. A two-sided *p*-value < 0.05 was considered statistically significant. All analyses were conducted using Stata/SE version 18 (StataCorp, College Station, TX, USA).

## 3. Results

Among the 307 patients included in the analysis, 69 patients (22%) underwent preoperative planning and intraoperative guidance using patient-specific 3D virtual models, while 238 patients (78%) constituted the control group and were treated using conventional 2D imaging modalities alone. Baseline demographic and clinical characteristics were well balanced between the two groups. No statistically significant differences were observed in median age (62 vs. 61 years, *p* = 0.5), BMI (28 vs. 26 kg/m^2^, *p* = 0.3), or baseline renal function as assessed by preoperative eGFR (85 vs. 86 mL/min/1.73 m^2^, *p* = 0.5).

The median tumor size was 4.2 cm (IQR 3.1–7.4) in the 3D group compared to 4.0 cm (IQR 2.9–7.2) in the control group (*p* = 0.4). These values are consistent with our pathological T-stage distribution (T1 67%, T2 30%, T3–T4 3.6%; *p* = 0.8), which includes a relevant proportion of T2 tumors. The RENAL nephrometry score was used as the sole metric for tumor complexity assessment and showed comparable distributions between the two cohorts (*p* = 0.12). Low-complexity tumors were observed in 75% of patients in the 3D group and 72% in the control group, intermediate-complexity tumors in 22% versus 28%, and high-complexity tumors in 2.9% versus 0.4%, respectively.

The conversion rate to radical nephrectomy was low in both cohorts, occurring in 5% of patients in the 3D group (3/69) and 2% in the control group (5/238), with no statistically significant difference (*p* = 0.1). Regarding the clamping strategy, selective or super-selective clamping was significantly more frequent in the 3D group compared to the control group (32% vs. 15%; *p* < 0.01). Main renal artery clamping (on-clamp) remained the standard for the most complex cases in both cohorts, while an off-clamp approach was utilized in 8% of the overall population.

The median WIT was 17.5 min (IQR 13–21) in the 3D-assisted group and 18.5 min (IQR 15–23) in the control group (*p* = 0.09). Although the 3D group showed a trend toward shorter WIT, the difference did not reach statistical significance in this series. The median operative time was 155 min (IQR 138–180) for the 3D group and 162 min (IQR 142–188) for the control group (*p* = 0.12). Median EBL was 130 mL vs. 150 mL (*p* = 0.2), and the median LOS was 3 days (IQR 2–4) for both cohorts (*p* = 0.7), reflecting a standardized post-operative recovery protocol.

Notably, the rate of positive surgical margins was significantly lower in the 3D group compared with the control group (5% vs. 15%; *p* = 0.030), suggesting improved oncological precision associated with 3D-assisted planning. No significant association was found between histopathological subtype and surgical outcomes (*p* = 0.3). Preservation of renal function, defined as retention of at least 90% of preoperative eGFR at discharge, was achieved in 65% of patients in the 3D group and 63% in the control group (*p* = 0.8). Overall postoperative complication rates were similar, occurring in 26% of patients in the 3D group and 29% in the control group (*p* = 0.7), with no significant differences in the distribution of complication severity.

Trifecta achievement was significantly more frequent among patients who underwent 3D-assisted RAPN. Specifically, 51% of patients in the 3D group met all three trifecta criteria compared with 32% in the control group (*p* = 0.004). On multivariable logistic regression analysis, after adjusting for age, BMI, and RENAL nephrometry score, the use of 3D modeling remained an independent predictor of trifecta achievement (OR 2.1, 95% CI 1.17–3.70; *p* = 0.013), as reported in Table 2.

**Table 2:** Multivariate logistic regression analysis evaluating predictors of trifecta achievement in patients undergoing RAPN.

Variable	OR (95% CI)	*p*-Value
Age	0.98 (0.96–1.01)	0.12
BMI	0.97 (0.92–1.02)	0.20
RENAL Complexity		
Low complexity (ref)		
Moderate complexity	0.74 (0.48–1.14)	0.17
High complexity	0.52 (0.28–1.01)	0.09
Use of 3D Model		
No (ref)		
Yes	2.1 (1.17–3.70)	0.013

## 4. Discussion

In this study, we found that virtual 3D reconstructions improved surgical outcomes in patients with localized RCC undergoing RAPN. Their use was associated with a 19% higher likelihood of achieving a surgical trifecta.

Our findings align with an expanding body of evidence supporting the clinical value of 3D-assisted surgical planning. A large multicenter UroCCR analysis including more than 600 patients demonstrated that the use of 3D models for preoperative planning and intraoperative guidance was associated with lower rates of major complications and improved renal functional preservation. Importantly, trifecta achievement was significantly higher in the 3D-assisted cohort compared with standard imaging alone [19].

Further supporting these results, a propensity-score-matched study evaluating 3D virtual models (3DVMs) in RAPN showed that, in patients with high-complexity tumors (PADUA ≥ 10), the use of 3DVMs independently protected against clinically significant eGFR decline at 12 months. The authors also observed higher rates of pure enucleation and reduced need for global ischemia when 3D mapping was available [20].

Another important contribution to the field is the development of perfusion-based 3D models. A recent study using Voronoi-based perfusion algorithms (PR-3DVMs) showed that these maps accurately delineate segmental arterial territories, enabling highly selective or super-selective clamping. Patients undergoing super-selective clamping exhibited minimal postoperative renal function decline on nuclear scintigraphy compared with those who required main artery clamping, highlighting the potential functional advantages of perfusion-guided RAPN [21].

Additionally, 3D “segmental” reconstructions have been shown to assist with selective artery clamping. In a retrospective cohort of 422 patients, the availability of these models increased the use of selective artery clamping and was associated with significantly better postoperative renal function compared with main renal artery clamping [22]. These data reinforce the idea that vascular-oriented 3D reconstructions may meaningfully influence surgical strategy and functional outcomes [3].

Emerging technologies such as “digital anatomical twins,” incorporating tissue-texture simulation using machine-learning algorithms, represent a further evolution of virtual surgical planning. In a recent pilot study, surgeons rated these next-generation models as more realistic than conventional 3D reconstructions, particularly regarding the depiction of vascular pedicles, tumor boundaries, and collecting system anatomy [13]. Although early, such innovations may further enhance surgical precision as they mature.

Still, not all studies show a clear improvement across all outcomes. For example, the introduction of a CT-based 3D resection process map (RPM) did not significantly increase trifecta achievement, although it markedly reduced severe postoperative complications (Clavien ≥ 3) [23]. These findings suggest that 3D technology may particularly improve safety rather than uniformly enhancing every metric of surgical “quality.”

Recent evidence has also emphasized that tumor morphology, rather than imaging modality alone, remains a strong determinant of postoperative outcomes. A study evaluating tumors protruding into the renal sinus showed that certain morphologies (e.g., complex hump-shaped protrusion) predicted trifecta failure independently of surgical planning tools [24]. This indicates that even advanced 3D modeling has limitations in overcoming intrinsic anatomical challenges.

A comprehensive review has also highlighted several challenges that currently limit widespread adoption of 3D-assisted navigation. These include intraoperative organ deformation, registration inaccuracy, high generation costs, and workflow integration issues [25]. Continued refinement of segmentation pipelines, improved automation, and AI-based deformation prediction models may help address these limitations in the near future.

In addition to technical refinement, the clinical value of 3D modeling should also be interpreted in the context of surgical standardization. By providing a shared and reproducible anatomical reference, 3D virtual models may reduce inter-surgeon variability in operative planning and execution, particularly in centers with heterogeneous experience levels. This aspect is especially relevant for complex renal tumors, where anatomical interpretation strongly influences intraoperative decision-making. Furthermore, 3D-assisted planning may facilitate multidisciplinary discussion and improve patient counseling by enabling clearer visualization of surgical complexity and anticipated risks [26].

The present study has several limitations. The retrospective design introduces potential selection bias, especially because 3D reconstruction was introduced at a later stage in the institutional workflow.

In addition to the inherent limitations of its retrospective design, the present study may be affected by temporal and selection bias. Because 3D modeling was introduced in the later years of the study period and its use was at the discretion of the operating surgeon, potential confounding by indication cannot be entirely excluded. Although baseline characteristics were balanced and multivariable adjustment was applied, unmeasured factors—such as surgeon experience, evolving perioperative protocols, or unrecorded anatomical complexity—may have influenced the outcomes. However, all surgeons involved were high-volume operators with stable experience levels during the study period, and no major modifications in perioperative management occurred, reducing the likelihood of temporal bias. Future analyses adjusting for surgical year or incorporating propensity score–based matching would further strengthen the robustness of these findings.

Another important aspect is the relatively low proportion of high-complexity renal tumors in both cohorts. Although this reflects the real-world case mix of a tertiary referral center, it may limit the generalizability of our findings to the most complex cases. Further investigations focusing on high PADUA or RENAL score lesions could better elucidate the potential incremental benefit of 3D modeling in technically demanding scenarios.

Moreover, renal function was assessed using eGFR at hospital discharge, which accurately represents perioperative preservation but does not account for delayed recovery or long-term function. The absence of standardized mid- or long-term follow-up (e.g., 3–12 months) represents a limitation that warrants future prospective evaluation.

Beyond perioperative metrics, future research should also assess medium- and long-term renal function, cost-effectiveness, and workflow feasibility of large-scale 3D model integration. Establishing standardized segmentation pipelines and automating the generation process could reduce time and costs, facilitating routine adoption in high-volume centers.

Overall, the evidence, including the findings from our study, suggests that 3D virtual models can meaningfully enhance surgical planning, increase operative precision, reduce complications, and potentially improve renal functional preservation in RAPN. As computational methods, automation, and artificial intelligence continue to advance, 3D modeling will likely become increasingly standardized and integral to the workflow of nephron-sparing surgery.

## 5. Conclusions

The integration of patient-specific 3D virtual modeling into the surgical workflow for robot-assisted partial nephrectomy was associated with improved perioperative outcomes, particularly through enhanced surgical margin control and higher trifecta achievement rates.

While these findings suggest potential advantages of 3D-assisted planning, the retrospective nature of the analysis and the non-randomized allocation preclude definitive conclusions regarding causality.

Future prospective and cost-effectiveness studies are warranted to confirm these associations, with particular attention to patients presenting high anatomical complexity and to long-term renal functional outcomes. As computational technologies and artificial intelligence continue to evolve, this approach is expected to become increasingly standardized, supporting greater precision and reproducibility in nephron-sparing surgery.

## Data Availability

The original contributions presented in this study are included in the article. Further inquiries can be directed to the corresponding author.

## Abbreviations

The following abbreviations are used in this manuscript:

RAPN	Robot-assisted partial nephrectomy
eGFR	Estimated glomerular filtration rate
BMI	Body mass index
